# Supplementary material for: Molecular dynamics simulations reveal membrane lipid interactions of the full-length lymphocyte specific kinase (Lck)
Source: Sci Rep. 2022 Dec 7;12:21121. doi: 10.1038/s41598-022-25603-6 (PMC9729596; doi:10.1038/s41598-022-25603-6)
Supplement: Supplementary file 2 — Supplementary Figures. [file 41598_2022_25603_MOESM2_ESM.pdf]

Molecular dynamics simulations reveal membrane lipid interactions of the full-length lymphocyte specific kinase Lck

Dheeraj Prakaash [1, 2], Charline Fagnen [1, 2], Graham P. Cook [3], Oreste Acuto [4] and Antreas C. Kalli [1, 2] *

[1] Leeds Institute of Cardiovascular and Metabolic Medicine, School of Medicine, University of Leeds, United Kingdom.
[2] Astbury Center for Structural Molecular Biology, University of Leeds, United Kingdom.
[3] Leeds Institute of Medical Research, School of Medicine, University of Leeds, United Kingdom.
[4] Sir William Dunn School of Pathology, University of Oxford, United Kingdom.

* Correspondence: a.kalli@leeds.ac.uk

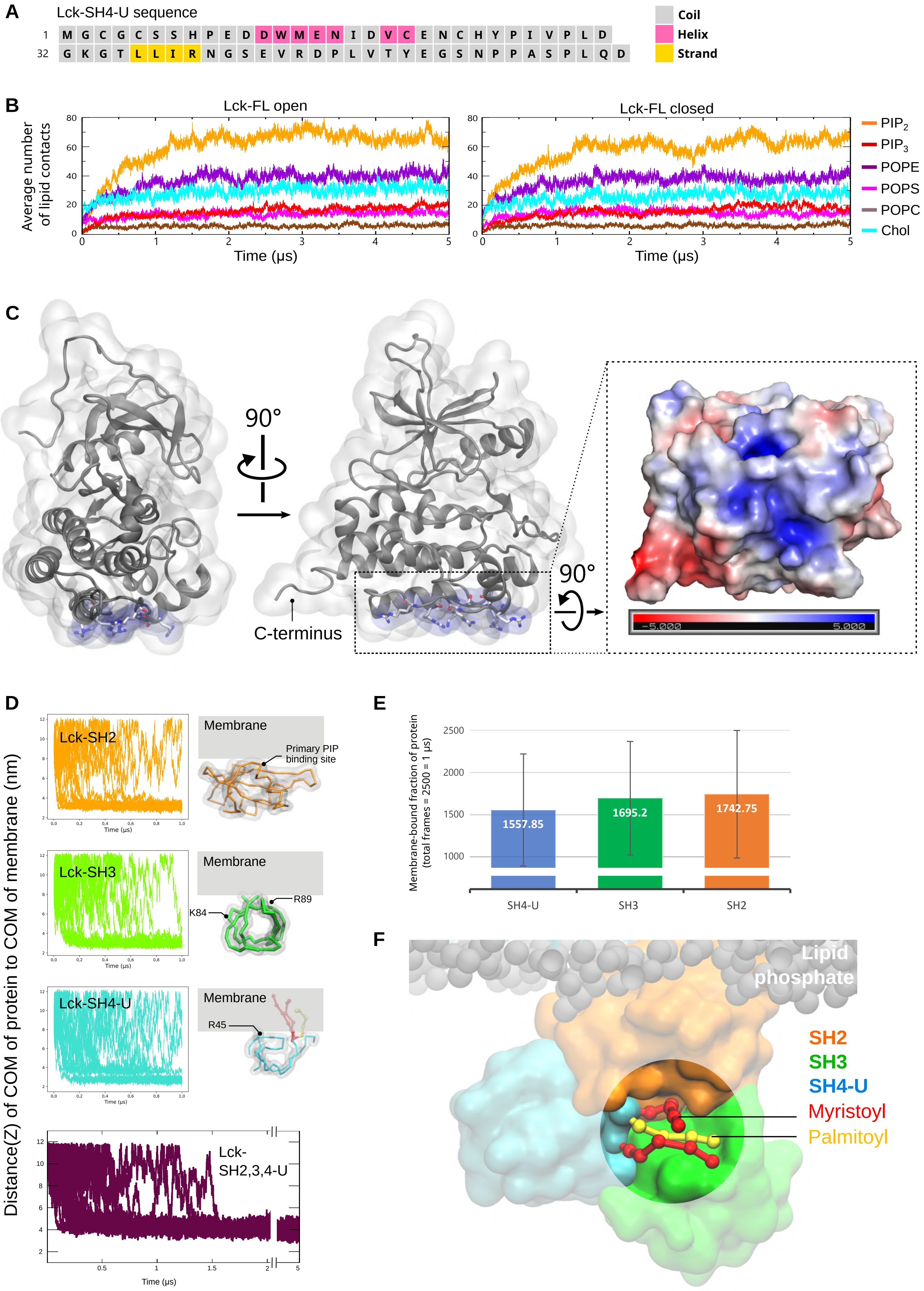
A
Lck-SH4-U sequence
1 M G C G C S S H P E D D W M E N I D V C E N C H Y P I V P L D
32 G K G T L L I R N G S E V R D P L V T Y E G S N P P A S P L Q D
Coil
Helix
Strand
B
Average number of lipid contacts
Lck-FL open
Lck-FL closed
Time (μs)
PIP2
PIP3
POPE
POPS
POPC
Chol
C
90°
C-terminus
-5.000
5.000
D
Distance(Z) of COM of protein to COM of membrane (nm)
Lck-SH2
Membrane
Primary PIP binding site
Lck-SH3
K84
R89
Lck-SH4-U
R45
Lck-SH2,3,4-U
Time (μs)
E
Membrane-bound fraction of protein (total frames = 2500 = 1 μs)
1557.85
1695.2
1742.75
SH4-U
SH3
SH2
F
Lipid phosphate
SH2
SH3
SH4-U
Myristoyl
Palmitoyl

Fig S1. Secondary structure prediction of Lck-SH4-U, membrane binding of individual Lck-SH domains, and electrostatics of the kinase domain. (A) Secondary structure prediction of Lck-SH4-U by PSIPRED 4.0 server. **(B)** Non-normalized number of contacts between Lck and lipid headgroups versus time averaged from all 20 CGMD simulations from the Lck-FL open (left) and Lck-FL closed simulations (right). **(C)** Residues in the kinase domain forming a flat cationic patch. Their electrostatic profile was calculated using in the ±5 kT/e range and at pH 7.0 using the PDB2PQR and APBS tools. Electronegative and electropositive regions are indicated by red and blue intensities respectively. **(D)** Distance between the center of mass (COM) of protein to COM of membrane in all 20 simulation replicates: SH4-U (cyan), SH3 (green), SH2 (orange), Lck-SH2,3,4-U (maroon). The most observed membrane-bound orientations of the individually simulated Lck-SH domains are also shown. Residues interacting most with the membrane in these simulations are labelled. **(E)** Average number of frames in the individual Lck-SH simulations that the protein stayed unbound (red) or bound (green) to the membrane. **(F)** A snapshot from one of the Lck-SH2,3,4-U simulations highlighting the binding pocket of the SH4 lipid tails (near the SH2-SH3 linker loop region) when they did not insert into the membrane.

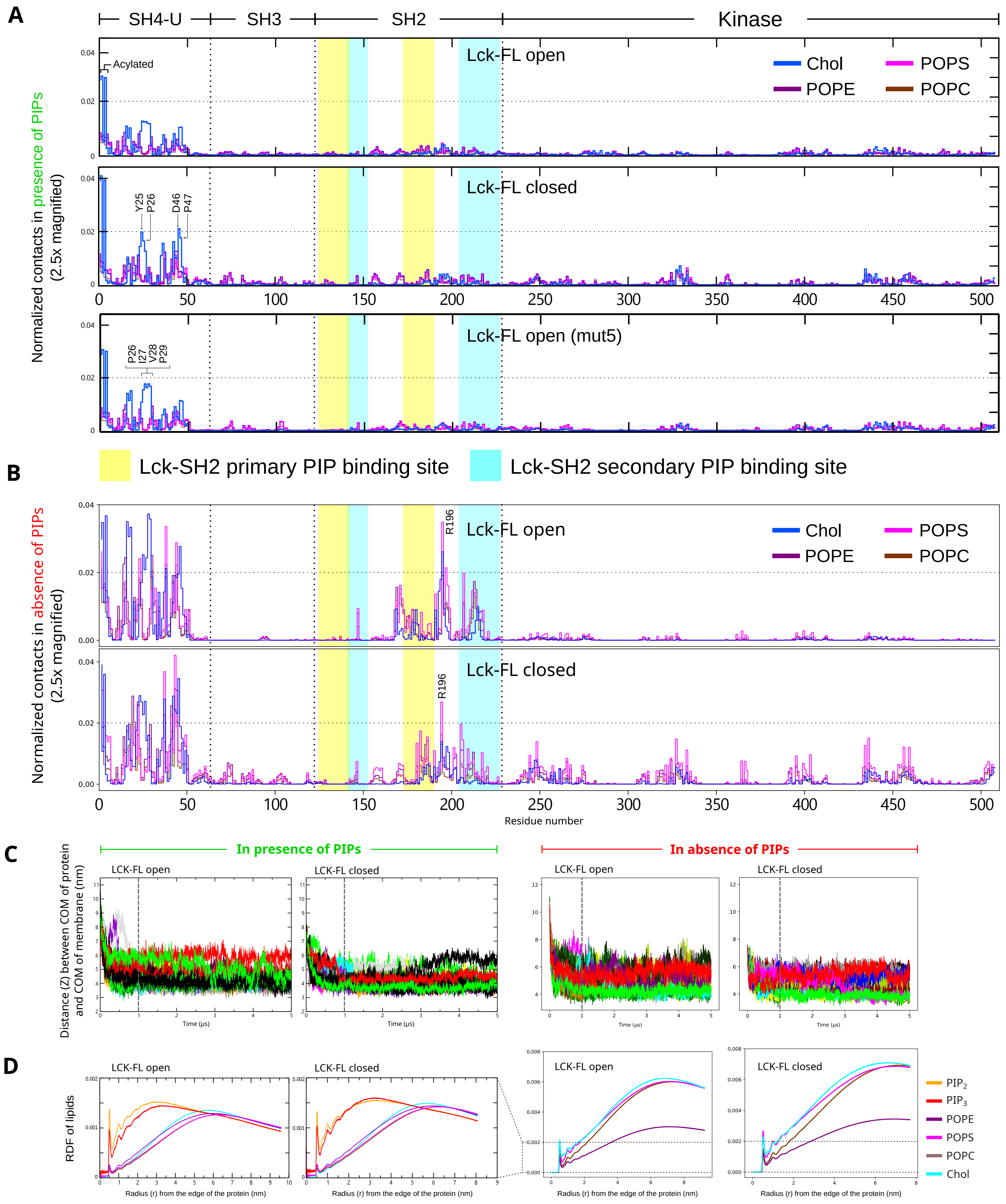
A
SH4-U
SH3
SH2
Kinase
Lck-FL open
Acylated
Chol
POPS
POPE
POPC
Lck-FL closed
Y25
P26
D46
P47
Lck-FL open (mut5)
P26
I27
V28
P29
Normalized contacts in presence of PIPs (2.5x magnified)
Lck-SH2 primary PIP binding site
Lck-SH2 secondary PIP binding site
B
R196
Normalized contacts in absence of PIPs (2.5x magnified)
Residue number
C
In presence of PIPs
In absence of PIPs
LCK-FL open
LCK-FL closed
Distance (Z) between COM of protein and COM of membrane (nm)
Time (μs)
D
RDF of lipids
Radius (r) from the edge of the protein (nm)
PIP2
PIP3
POPE
POPS
POPC
Chol

Fig S2. A comparison of lipid interactions of Lck-FL open and closed conformations in the presence and absence of PIP lipids in the membrane. (A) Normalized lipid interactions calculated from the Lck-FL open, closed, and open (mut5) simulations in the presence of PIP lipids and **(B)** absence of PIP lipids in the membrane. **(C)** Distance between the center of mass (COM) of Lck-FL and COM of the membrane versus time in the presence (left) and absence of PIPs (right) in the membrane. The line drawn at 1 μs is to indicate that Lck-FL binds to the membrane by this point in time in all simulations. **(D)** The normalized radial distribution function (RDF) of all lipid types around Lck-FL calculated throughout the simulation time from all 20 CGMD simulations combined. The RDF data enables comparison between Lck-FL open and closed conformations, and also between the presence and absence of PIPs in the membrane. The RDF is normalized by the total number of lipids in the membrane of that system to enable comparison between the open and closed conformations of Lck-FL.

A SH2 orientation (Lck-FL open)
(all 20 MD simulation replicates combined)

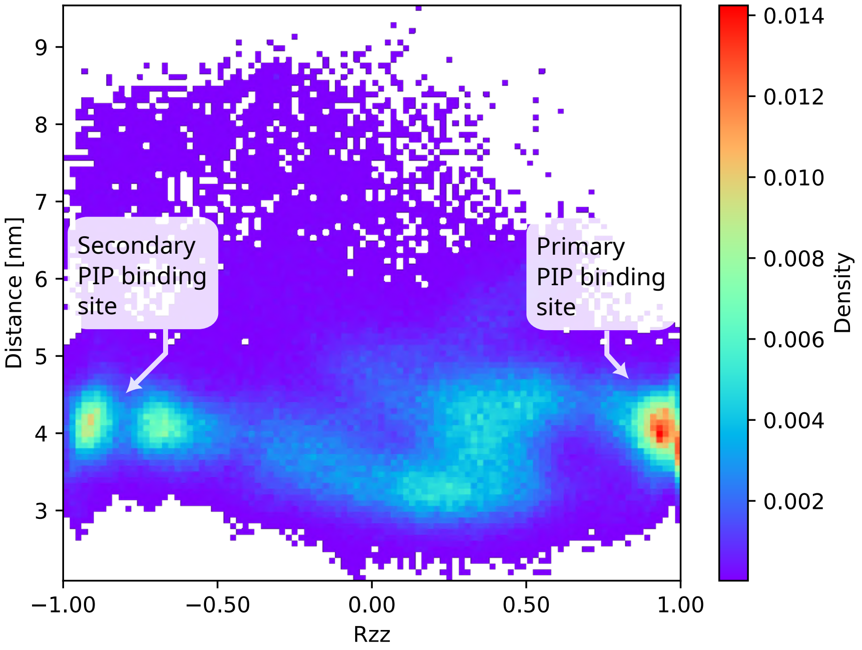

B SH2 orientation (Lck-FL open)
(all 20 MD simulation replicates individually analysed)

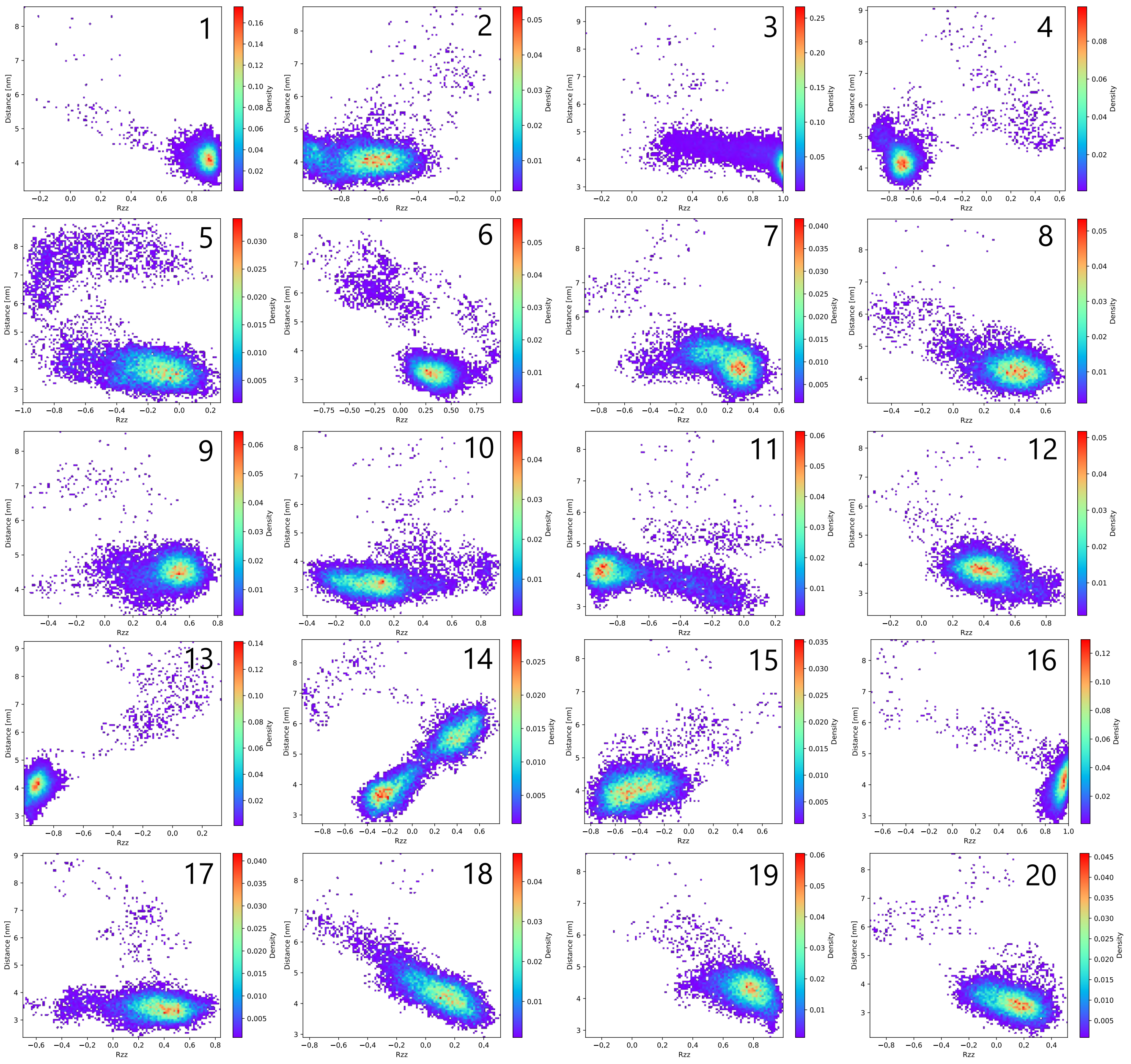

Fig S3. The membrane-bound orientation of the Lck-SH2 domain in the Lck-FL open conformation in the presence of PIPs in the membrane (A) as shown from all CGMD simulations combined and **(B)** in each CGMD simulation replicate individually. In all these plots, the X axis represents the ZZ component of the rotational matrix of the protein, and the Y axis represents the distance between the center of mass of the protein and the center of mass of the membrane along the Z axis (perpendicular to the membrane).

A

SH2 orientation (Lck-FL closed)
(all 20 MD simulation replicates combined)

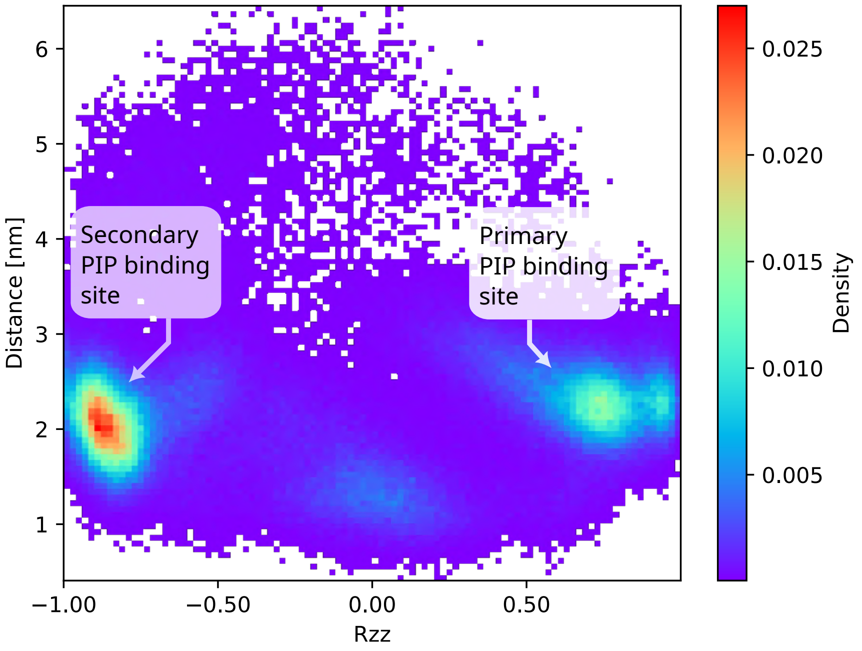

B

SH2 orientation (Lck-FL closed)
(all 20 MD simulation replicates individually analysed)

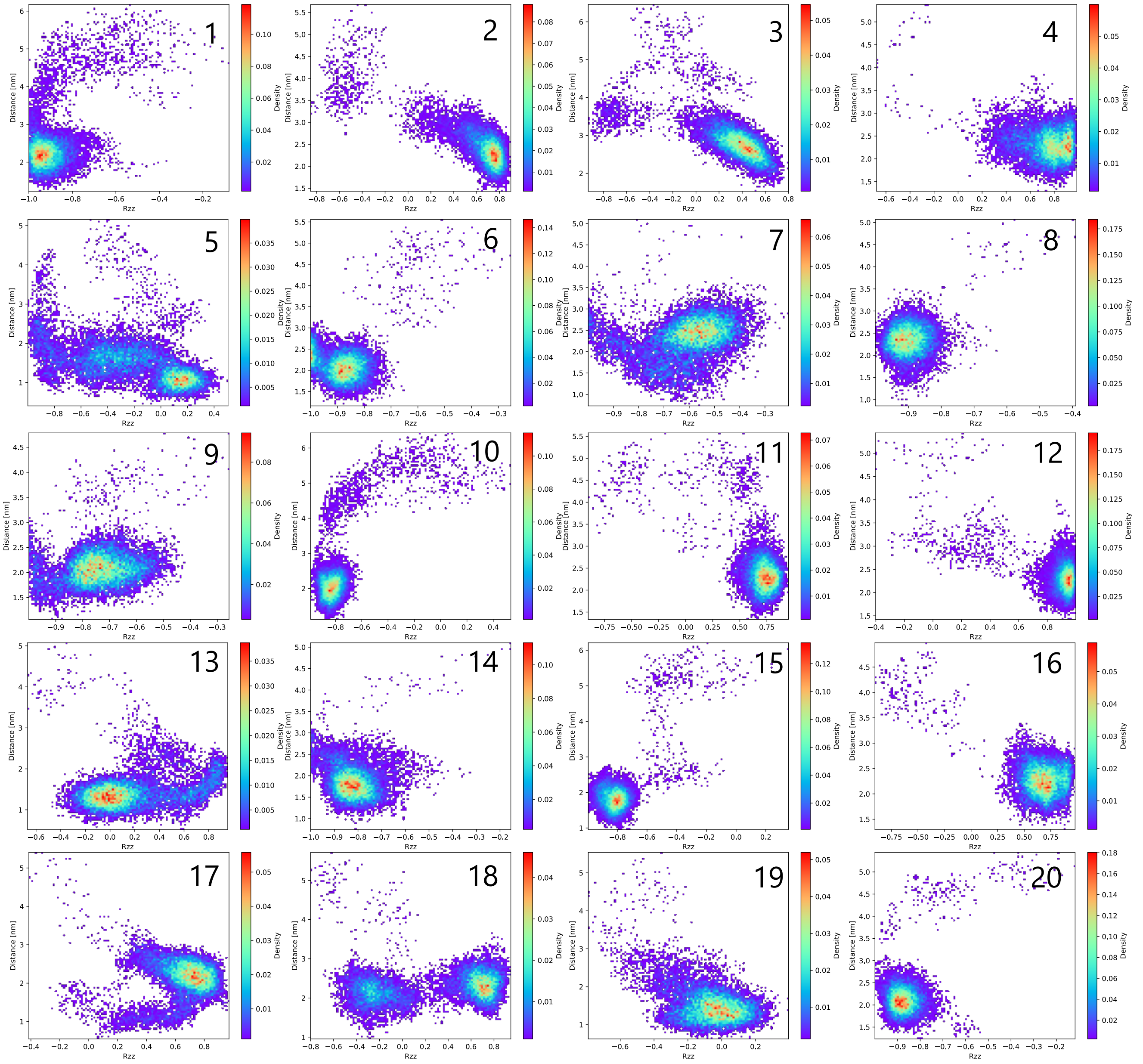

Fig S4. The membrane-bound orientation of the Lck-SH2 domain in the Lck-FL closed conformation in the presence of PIPs in the membrane (A) as shown from all CGMD simulations combined and **(B)** in each CGMD simulation replicate individually. In all these plots, the X axis represents the ZZ component of the rotational matrix of the protein, and the Y axis represents the distance between the center of mass of the protein and the center of mass of the membrane along the Z axis (perpendicular to the membrane).

A

```
CLUSTAL O(1.2.4) Multiple Sequence Alignment


P06241|Fyn      ---------------------GCVQCKDKEATKLTEERDGSLN---QSSGYRYGTDPTPQ 36
P12931|Src      ---------------------GSNKSKPKDASQ----RRRSLEPAENVHGAGGGAFPASQ 35
P07947|Yes      ---------------------GCIKSKENKSPAIKYRPENTPEPVSTS-VSHYGAEPTTV 38
P06239|Lck      ---------GC----------GCSSHPEDDW-------MENIDVCENCHYPIVPLDGK-- 32
P08631|Hck      GGRSSCEDPGCPRDEERAPRMGCMKSKFLQVGG--N-TFSKTETSASPHCPVYVPDPT-- 55
P25911|Lyn      ---------------------GCIKSKRKDNLNDDE-VDSKTQPVRNTDRTIYVRDPT-- 36
                                     *. .    .          . :

P06241|Fyn      HYPSF-------GV-TSIPNYNNFHAAGGQGLTVFGGVNSSSHTGTLRT----RGGTGVT 84
P12931|Src      TPSKPASADGHRGPSAAFAPA-------AAEPKLFGGFNSSDTVTSPQR--AGPLAGGVT 86
P07947|Yes      SPCP---SSSAKGTAVNFSSLSMTPFGGSSGVTPFGGASSSFSVVPSSY--PAGLTGGVT 93
P06239|Lck      -----------------------------GTLLIRNGSEVRDPLVTYEGSNPPASPLQDN 63
P08631|Hck      -----------------------------STIK--PGPNSHNSNTPGIR----EAGSEDI 80
P25911|Lyn      -----------------------------SNKQ--QRPVPEFHLLPGQRFQTKDPEEQGD 65


P06241|Fyn      LFVALYDYEARTEDDLSFHKGEKFQILNSSEGDWWEARSLTTGETGYIPSNYVAPVDSIQ 144
P12931|Src      TFVALYDYESRTETDLSFKKGERLQIVNNTEGDWWLAHSLSTGQTGYIPSNYVAPSDSIQ 146
P07947|Yes      IFVALYDYEARTTEDLSFKKGERFQIINNTEGDWWEARSIATGKNGYIPSNYVAPADSIQ 153
P06239|Lck      LVIALHSYEPSHDGDLGFEKGEQLRILE-QSGEWWKAQSLTTGQEGFIPFNFVAKANSLE 122
P08631|Hck      IVVALYDYEAIHHEDLSFQKGDQMVVLE-ESGEWWKARSLATRKEGYIPSNYVARVDSLE 139
P25911|Lyn      IVVALYPYDGIHPDDLSFKKGEKMKVLE-EHGEWWKAKSLSSKREGFIPSNYVAKVNTLE 124
                 .:**: *:     **.*.**::: :::   *:** *:*::: . *:** *:**  ::::

P06241|Fyn      AEEWYFGKLGRKDAERQLLSFGNPRGTFLIRESETTKGAYSLSIRDWDDMKGDHVKHYKI 204
P12931|Src      AEEWYFGKITRRESERLLLNAENPRGTFLVRESETTKGAYCLSVSDFDNAKGLNVKHYKI 206
P07947|Yes      AEEWYFGKMGRKDAERLLLNPGNQRGIFLVRESETTKGAYSLSIRDWDEIRGDNVKHYKI 213
P06239|Lck      PEPWFFKNLSRKDAERQLLAPGNTHGSFLIRESESTAGSFSLSVRDFDQNQGEVVKHYKI 182
P08631|Hck      TEEWFFKGISRKDAERQLLAPGNMLGSFMIRDSETTKGSYSLSVRDYDPRQGDTVKHYKI 199
P25911|Lyn      TEEWFFKDITRKDAERQLLAPGNSAGAFLIRESETLKGSFSLSVRDYDPMHGDVIKHYKI 184
                 * *:*  : *:::** **   *   * *::*:**:  *::.**: *:*  :*  :*****

P06241|Fyn      RKLDNGGYYITTRAQFETLQQLVQHYSERAAGLCCRLVVPCHKGMPRLTDLSVKTKDVWE 264
P12931|Src      RKLDSGGFYITSRTQFNSLQQLVAYYSKHADGLCHRLTTVCPTSKPQTQGL---AKDAWE 263
P07947|Yes      RKLDNGGYYITTRAQFDTLQKLVKHYTEHADGLCHKLTTVCPTVKPQTQGL---AKDAWE 270
P06239|Lck      RNLDNGGFYISPRITFPGLHELVRHYTNASDGLCTRLSRPCQTQKPQKPW----WEDEWE 238
P08631|Hck      RTLDNGGFYISPRSTFSTLQELVDHYKKGNDGLCQKLSVPCMSSKPQKPW----EKDAWE 255
P25911|Lyn      RSLDNGGYYISPRITFPCISDMIKHYQKQSDGLCRRLEKACISPKPQKPW----DKDAWE 240
                *.**.**:**: *  *  : .:: :* :   *** :*   * .  *:        :* **

P06241|Fyn      IPRESLQLIKRLGNGQFGEVWMGTWNGNTKVAIKTLKPGTMSPESFLEEAQIMKKLKHDK 324
P12931|Src      IPRESLRLEVKLGQGCFGEVWMGTWNGTTRVAIKTLKPGTMSPEAFLQEAQVMKKLRHEK 323
P07947|Yes      IPRESLRLEVKLGQGCFGEVWMGTWNGTTKVAIKTLKPGTMMPEAFLQEAQIMKKLRHDK 330
P06239|Lck      VPRETLKLVERLGAGQFGEVWMGYYNGHTKVAVKSLKQGSMSPDAFLAEANLMKQLQHQR 298
P08631|Hck      IPRESLKLEKKLGAGQFGEVWMATYNKHTKVAVKTMKPGSMSVEAFLAEANVMKTLQHDK 315
P25911|Lyn      IPRESIKLVKKLGAGQFGEVWMGYYNNSTKVAVKTLKPGTMSVQAFLEEANLMKTLQHDK 300
                :***:::*  :** * ******. :*  *:**:*::* *:*  ::** **::** *:*::

P06241|Fyn      LVQLYAVVS-EEPIYIVTEYMNKGSLLDFLKDGEGRALKLPNLVDMAAQVAAGMAYIERM 383
P12931|Src      LVQLYAVVS-EEPIYIVTEYMSKGSLLDFLKGETGKYLRLPQLVDMAAQIASGMAYVERM 382
P07947|Yes      LVPLYAVVS-EEPIYIVTEFMSKGSLLDFLKEGDGKYLKLPQLVDMAAQIADGMAYIERM 389
P06239|Lck      LVRLYAVVT-QEPIYIITEYMENGSLVDFLKTPSGIKLTINKLLDMAAQIAEGMAFIEER 357
P08631|Hck      LVKLHAVVT-KEPIYIITEFMAKGSLLDFLKSDEGSKQPLPKLIDFSAQIAEGMAFIEQR 374
P25911|Lyn      LVRLYAVVTKEEPIYIITEFMAKGSLLDFLKSDEGGKVLLPKLIDFSAQIAEGMAYIERK 360
                ** *:***: :*****:**:* :***:****   *    : :*:*::**:* ***::*.

P06241|Fyn      NYIHRDLRSANILVGNGLICKIADFGLARLIEDNEYTARQGAKFPIKWTAPEAALYGRFT 443
P12931|Src      NYVHRDLRAANILVGENLVCKVADFGLARLIEDNEYTARQGAKFPIKWTAPEAALYGRFT 442
P07947|Yes      NYIHRDLRAANILVGENLVCKIADFGLARLIEDNEYTARQGAKFPIKWTAPEAALYGRFT 449
P06239|Lck      NYIHRDLRAANILVSDTLSCKIADFGLARLIEDNEYTAREGAKFPIKWTAPEAINYGTFT 417
P08631|Hck      NYIHRDLRAANILVSASLVCKIADFGLARVIEDNEYTAREGAKFPIKWTAPEAINFGSFT 434
P25911|Lyn      NYIHRDLRAANVLVSESLMCKIADFGLARVIEDNEYTAREGAKFPIKWTAPEAINFGCFT 420
                **:*****:**:**.  * **:*******:*********:*************  :* **

P06241|Fyn      IKSDVWSFGILLTELVTKGRVPYPGMNNREVLEQVERGYRMPCPQDCPISLHELMIHCWK 503
P12931|Src      IKSDVWSFGILLTELTTKGRVPYPGMVNREVLDQVERGYRMPCPPECPESLHDLMCQCWR 502
P07947|Yes      IKSDVWSFGILQTELVTKGRVPYPGMVNREVLEQVERGYRMPCPQGCPESLHELMNLCWK 509
P06239|Lck      IKSDVWSFGILLTEIVTHGRIPYPGMTNPEVIQNLERGYRMVRPDNCPEELYQLMRLCWK 477
P08631|Hck      IKSDVWSFGILLMEIVTYGRIPYPGMSNPEVIRALERGYRMPRPENCPEELYNIMMRCWK 494
P25911|Lyn      IKSDVWSFGILLYEIVTYGKIPYPGRTNADVMSALSQGYRMPRMENCPDELYDIMKMCWK 480
                ***********  *:.* *::****  * :*:  :.:****     ** .*:::*  **:

P06241|Fyn      KDPEERPTFEYLQSFLEDYFTATEPQYQPGENL 536
P12931|Src      KEPEERPTFEYLQAFLEDYFTSTEPQYQPGENL 535
P07947|Yes      KDPDERPTFEYIQSFLEDYFTATEPQYQPGENL 542
P06239|Lck      ERPEDRPTFDYLRSVLEDFFTATEGQYQPQP-- 508
P08631|Hck      NRPEERPTFEYIQSVLDDFYTATESQYQQQP-- 525
P25911|Lyn      EKAEERPTFDYLQSVLDDFYTATEGQYQQQP-- 511
                :  ::****:*:::.*:*::*:** ***
```

SH2 domain

Kinase domain

primary PIP binding site (SH2)

secondary PIP binding site (SH2)

significant contacts with PIPs (Kinase)

potential contact sites with PIPs (Kinase)

(Lck-PIP interactions were observed via these sites but not as significantly as those highlighted in magenta)

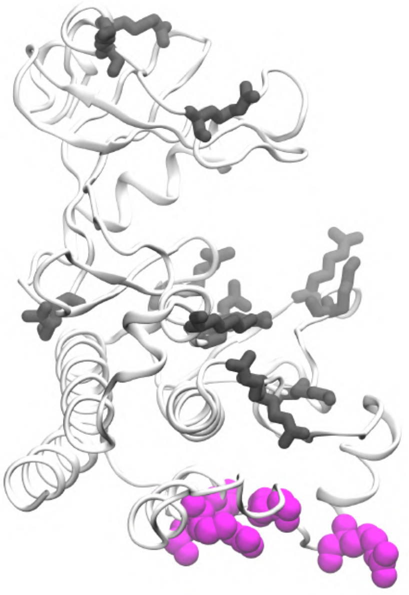

B

Percent Identity Matrix - created by Clustal2.1

Phylogenetic tree

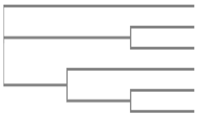

	Fyn	Src	Yes	Lck	Hck	Lyn
P06241|Fyn	100.00	70.88	73.63	57.52	60.48	57.65
P12931|Src	70.88	100.00	75.52	54.76	58.05	54.65
P07947|Yes	73.63	75.52	100.00	56.26	62.03	57.20
P06239|Lck	57.52	54.76	56.26	100.00	**66.33**	63.49
P08631|Hck	60.48	58.05	62.03	66.33	100.00	72.22
P25911|Lyn	57.65	54.65	57.20	63.49	72.22	100.00

Fig S5. Multiple sequence alignment and the identity of the full-length Lck with other members of the Src family of kinases. (A) Multiple sequence alignment, obtained by Clustal Omega, highlighting the residues in the SH2 and kinase domains that mediated Lck-PIP interactions. These residues are observed to be conserved. Note that the myristoylated G2 residue is considered as the first residue, hence all residues are shifted one position behind i.e., 'n-1'. Refer Fig 3E to visualize the primary and secondary PIP lipid binding sites of Lck-SH2. The figure shown on the right is a cartoon representation of the kinase domain with significant PIP contacting residues shown as magenta spheres, and potentially contacting residues shown as grey sticks. **(B)** Schematic phylogenetic tree showing the evolution of Src family of kinases and their identity matrix (calculated in %). This suggests that Lck is most similar to Hck compared to other Src family members. The protein sequences were obtained from Uniprot (whose IDs are listed beside the respective protein names).

Movies

S1 Movie. CGMD simulation displaying insertion of myristoylated and palmitoylated lipid tails of the SH4 domain into the membrane. Phospholipid tails are shown as transparent grey spheres and their headgroups as transparent coloured spheres (POPC: brown, POPE: purple, PIP_2: orange, PIP_3: red). The myristoylated and palmitoylated residues are shown as yellow and red ball and sticks respectively. Lck-SH4-U backbone is shown as white bonds, and its PIP lipid binding residues R39, R45 are shown as blue surfaces.
